# Sexual selection and speciation: a meta-analysis of comparative studies

**DOI:** 10.1093/evlett/qraf038

**Published:** 2025-10-22

**Authors:** Tim Janicke, Tamra C Mendelson, Michael G Ritchie, Lucas Marie-Orleach, Jeanne Tonnabel

**Affiliations:** CEFE, Univ Montpellier, CNRS, EPHE, IRD, Montpellier, France; Department of Biological Sciences, University of Maryland, Baltimore County, Baltimore, MD, United States; Centre for Biological Diversity, School of Biology, University of St Andrews, St Andrews, United Kingdom; Institut de Recherche sur la Biologie de l’Insecte, UMR 7261, CNRS, Université de Tours, Tours, France; ISEM, University Montpellier, CNRS, IRD, Montpellier, France

**Keywords:** competition for mates, mate choice, speciation, species richness

## Abstract

Understanding the drivers of biodiversity is a central goal in evolutionary biology. In particular, sexual selection has long been proposed as a potential catalyst of speciation, but empirical evidence remains inconclusive. Here, we present a comprehensive meta-analysis synthesizing 145 effect sizes from 50 comparative studies testing the relationship between proxies of sexual selection and species diversity across the animal kingdom. Our results reveal a modest but consistent positive association (global effect size: *r* = 0.201; 95% confidence interval: 0.035–0.366), supporting the hypothesis that sexual selection contributes to speciation. However, the global effect size corresponds to an *R*^2^ of only 0.04, suggesting that sexual selection is not a dominant driver of speciation. We also uncover substantial heterogeneity among effect sizes, largely attributable to between-study variation and taxonomic affinities of effect sizes. Studies that fail to account for phylogenetic non-independence tend to report stronger effects. In contrast, other tested methodological and biological moderators, such as the proxies used to estimate the strength of sexual selection or proxies of speciation, do not explain the observed heterogeneity in effect sizes. Sensitivity analyses confirm the robustness of our results, and we find no signatures of publication bias. We highlight the need for broader taxonomic coverage and a greater focus on understudied mechanisms, such as post-copulatory sexual selection, to refine our understanding of the role of sexual selection in shaping species diversity.

## Introduction

Understanding the origin of the vast diversity of life forms lies at the heart of evolutionary biology. When Darwin laid the foundations of the modern theory of ecological speciation, he also introduced a distinctive form of natural selection that he termed sexual selection (Darwin, 1859, 1871). He observed that traits associated with mate choice and male–male competition often differ strikingly among closely related taxa, which may have spurred the idea that sexual selection can drive speciation (West-Eberhard, 1983). However, the conceptual framework linking species diversity and sexual selection was developed and explored much later.

Fisher’s concept of runaway sexual selection (Fisher, 1930) was the precursor for Lande’s influential quantitative genetic models, which demonstrated how the coevolution of mating preferences and display traits can drive speciation (Lande, 1981, 1982). Building on this framework, much of the subsequent theoretical work has emphasized the role of mate choice, particularly assortative mating, in promoting reproductive isolation (Higashi et al., 1999; van Doorn et al., 2009). Other mechanisms, such as competition for mates (Dijkstra & Border, 2018; Tinghitella et al., 2018) and sexual conflict (Gavrilets & Waxman, 2002; Parker & Partridge, 1998), have also been proposed as powerful forces shaping species divergence. Yet, despite the intuitive appeal of sexual selection as a driver of speciation, an opposing strand of theory highlights its potential to constrain divergence. For instance, Fisherian sexual selection can counteract local adaptation when gene flow occurs between populations (Servedio & Burger, 2014), and assortative mating may generate stabilizing selection if individuals with rare genotypes face reduced mating success ultimately impeding sympatric speciation (Kirkpatrick & Nuismer, 2004). Moreover, although sexual signals can diverge rapidly in allopatry, assortative mating can easily break down upon secondary contact, preventing continued reproductive isolation (Mendelson et al., 2014). The increasing complexity of this body of theoretical work has made it difficult even for experts to keep track of key developments (Weissing et al., 2011). Yet, most researchers seem to agree that the theoretical support for the role of sexual selection in speciation remains ambiguous (Grether, 2019; Servedio, 2012), and that the conditions under which sexual selection can lead to speciation appear far more restrictive than early arguments and models initially suggested (Servedio & Boughman, 2017).

In accordance to the theoretical work, empirical evidence coming from case studies, experimental evolution experiments, and comparative approaches also provided mixed results. Probably the most compelling evidence stems from case studies on reproductive isolation among diverging populations or incipient species (Mendelson & Safran, 2021; Schield et al., 2024). Echoing the emphasis in the existing body of theoretical work, the vast majority of those studies focused on how mate choice can contribute to divergence. However, numerous mechanisms have also been proposed for the role of competition for mates and/or their gametes in promoting reproductive isolation (Lackey et al., 2018; Tinghitella et al., 2018). Lackey et al., (2024) conducted a systematic review, showing that 68% of all identified studies found support for a positive effect of male competition on divergence at the microevolutionary scale. A much less commonly used, yet powerful, approach involves experimental evolution studies designed to establish a causal link between sexual selection and speciation (White et al., 2020; Wiberg et al., 2021). Finally, another line of empirical evidence, on which we are focusing here, concerns comparative studies that aim to test whether proxies of sexual selection predict species diversity or speciation rates across broader taxonomic scales (ranging from comparisons among subspecies to broad-scale studies covering major classes across the animal kingdom). These studies generally do not distinguish between the two mechanisms through which sexual selection may promote species diversity: directly, by generating trait divergence and reproductive isolation (see above), and indirectly, by shaping population demography, which in turn affects species persistence over evolutionary time scales. With respect to the latter mechanism, sexual selection has been argued to facilitate the purging of deleterious alleles more efficiently, allowing species under strong sexual selection to adapt more rapidly and to persist longer under changing environmental conditions (Rowe & Rundle, 2021; Whitlock & Agrawal, 2009). Conversely, sexual selection can also promote sexual conflict, which may hinder adaptation (intra-locus sexual conflict) or impose significant demographic costs on a population (inter-locus sexual conflict) (Arnqvist & Rowe, 2005; Bonduriansky & Chenoweth, 2009; Flintham et al., 2023; Rankin et al., 2011).

By taking a meta-analytic approach, Kraaijeveld et al., (2011) synthesized the findings of comparative studies and provided evidence that sexual selection is typically positively associated with species diversity across the tested animal taxa. While being an influential contribution to the field, this study was subject to at least two critical limitations. First, the number of available studies at the time of publication was low such that the meta-analysis was based on 64 effect sizes from only 20 studies. This limited sample size also resulted in biased taxonomic coverage, with birds being heavily overrepresented. Second, the available statistical toolkit for meta-analyses at the time precluded robust correction for phylogenetic non-independence among included effect sizes, an issue widely recognized as essential for unbiased inference (Chamberlain et al., 2012; Cinar et al., 2022).

Here, we build on the earlier work of Kraaijeveld et al., (2011) and leverage recent advances in the field to provide a more robust test of the hypothesis that sexual selection affects speciation, as inferred from comparative studies across the animal tree of life. We also aim to explore how biological moderators influence the role of sexual selection in speciation, and how methodological moderators affect the likelihood of detecting its signal. Finally, our systematic literature search seeks to identify methodological biases and knowledge gaps in comparative studies of sexual selection and speciation.

## Methods

### Systematic literature search

We followed the PRISMA guidelines (Moher et al., 2009; O’Dea et al., 2021) and conducted a systematic literature search to identify comparative studies testing for a relationship between proxies of sexual selection and speciation. Specifically, we searched for primary studies in Web of Science Core Collection and Scopus databases on April 20th, 2024 (updated on February 10th, 2025) with strings defined as “((“sexual selection” OR “sexual*” OR “sperm comp*” OR “ornament” OR “armament”) AND (“speciation” OR “diversification” OR “species richness” OR “extinction”))” screening “All Fields” (Web of Science) or “TITLE-ABS-KEY“ (Scopus). After deduplication using the ASySD R-package (Hair et al., 2023) this database search identified 10,202 references of which 9,943 could be excluded based on title and abstract screening (equally shared among T.J., L.M.O., and J.T.). We examined the full texts of the remaining 259 references to determine whether they met our sole inclusion criterion, which was that the study tested for a relationship between any proxy of sexual selection and any proxy of speciation (e.g., species richness, speciation rate) using a comparative method. Thus, whenever the authors of a comparative study considered their work as a test of the hypothesis that sexual selection affects speciation, we retained their study. This search yielded 46 primary studies of which two had to be excluded because we could not extract effect sizes based on the provided information, and we failed to get in contact with the authors. The resulting 44 primary studies covered all but one reference that was included in the previous meta-analysis by Kraaijeveld et al., (2011). We complemented our systematic literature search by backward searches of references mentioned in all identified primary studies and 20 selected key reviews on the topic (Supplementary Text S1), which resulted in one additional study. Moreover, we ran a forward search for references that are citing the previous meta-analysis by Kraaijeveld et al., (2011) in GoogleScholar on 10th of February 2025, which allowed us to identify four additional primary studies. Finally, we posted requests for unpublished data on the (academic) social media platforms ResearchGate, X, and BlueSky, which did not increase our sample size. Therefore, our systematic literature search identified a total of 50 primary studies eligible for inclusion in our meta-analysis (for PRISMA diagram see Figure S1; Supplementary Text S2), more than doubling the sample size of the previous meta-analysis.

### Phylogeny of sampled taxa

To account for phylogenetic non-independence, we assembled a time-calibrated phylogeny of the taxonomic groups covered in the primary studies by extracting divergence times from the TimeTree database (Kumar et al., 2022). Because this meta-analysis incorporates effect sizes derived from different taxonomic ranks, we nested lower-level taxa within higher-level taxa following a previously applied approach (Freitas et al., 2025; Winkler et al., 2024). For example, some primary studies on birds focused on comparisons within certain families, while others conducted analyses at the class level, encompassing multiple families. In these cases, we used the crown age, obtained from the TimeTree database, of the higher-level taxon (e.g., birds) to define its “divergence time” from the lower-level taxon (e.g., passerines). It is important to note that this approach is intended only to statistically control for phylogenetic non-independence of the compiled effect sizes. However, it has inherent limitations in terms of the biological interpretability of the resulting phylogenetic tree, since higher-level taxonomic ranks (e.g., birds) do not represent true outgroups for lower-level taxonomic ranks (e.g., passerines). The obtained matrix of divergence times between all partially nested taxa was then converted into the NEWICK format and finally into a correlation matrix using the *ape* R package (Paradis & Schliep, 2019).

### Statistical analysis

We used the Pearson correlation coefficient *r* as the target effect size of this meta-analysis. Statistics provided in the primary studies were converted into *r* and its sampling variance applying guidelines by Lajeunesse et al., (2013) and the *esc* R package (Lüdecke, 2019). For completeness, we also report key results using the Fisher’s *z* as an alternative effect size in the Supplementary Material. In total, we extracted 145 effect sizes from the 50 primary studies.

We fitted multi-level mixed effect models using the rma.mv function in *metafor* R package (Viechtbauer, 2010), with observation identifier (i.e., a unique number of each effect size), study identifier, taxon identifier, and the phylogeny defined in all models as random terms to account for non-independence of the sampled effect sizes. The global effect size, which quantifies the overall strength of the correlation between sexual selection and speciation proxies, was obtained from a null model including only the above-mentioned random terms but no fixed effects. In addition, we tested a set of biological and methodological moderators (Table S1). Specifically, we examined whether effect sizes differed among taxonomic classes and the speciation proxies used (i.e., species richness, diversification rate, and speciation rate). Furthermore, we used the proxy for the strength of sexual selection provided in the primary studies to classify four additional biological moderators (detailed in Table S2): the sexual selection proxy (i.e., presence of a sexually selected trait, mating system, sexual dichromatism, and sexual size dimorphism), the sexual selection mechanism (i.e., competition or choice), the mating stage (i.e., pre- or post-mating sexual selection), and the sex subject to sexual selection according to the proxy used (i.e., female, male, or both). Not all effect sizes could be associated with one of the sexual selection proxies and were therefore categorized as “Other”, including the expression of colonial breeding (*N* = 1), the presence of a sexual conflict trait (*N* = 1), spermathecal width (*N* = 1), and standardized selection metrics such as the Bateman gradient (*N* = 5), the opportunity for sexual selection (*N* = 6), the sex difference in the Bateman gradient (*N* = 1), the sex difference in the opportunity for sexual selection (*N* = 1), and the sex difference in the opportunity for selection (*N* = 1). We further tested whether correction for phylogenetic non-independence, as implemented in the primary studies (i.e., whether the primary study accounted for phylogenetic non-independence), explained heterogeneity among effect sizes. Each moderator was tested separately by adding it as a fixed effect to the null model.

All statistical analyses were run in R (R Core Team, 2024). Orchard plots visualizing the effects of moderators were generated with the help of the *orchard* R package (Nakagawa et al., 2023).

### Sensitivity analysis

We explored the robustness of our results by re-running the main analysis after excluding a subset of effect sizes based on three criteria: (i) effect sizes computed on an overly low sample size of *N* < 10 as they are subject to heightened sampling error (*N* = 4), (ii) effect sizes that have been identified as outliers based on Grubbs test using the *outliers* R-package (Komsta, 2022) (*N* = 1), and (iii) effect sizes that have a high Cook’s distance with a cut-off value of *D*_i_ > 4/N (Altman & Krzywinski, 2016) (*N* = 1). Thus, this more conservative analysis excludes in total six effect sizes from four primary studies (Table S3).

In addition, we examined the sensitivity of our findings with respect to the number of sampled studies obtained, which is admittedly still moderate. To do this, we visually explored how the observed global effect size changed over time with the continued publication of new studies in almost every year since Barraclough et al., (1995), which marked the first comparative study testing for a relationship between sexual selection and speciation (here inferred from a correlation between the proportion of sexually dichromatic species and species richness in passerine birds). Specifically, we assessed whether the global effect size remained stable despite the continued influx of new studies, in line with the expectation that it should have stabilized in recent years due to reduced sampling error as a result of increased sample sizes.

### Publication bias

We used multilevel meta-regression to assess potential publication bias based on the relationship between the effect size and its precision (Nakagawa et al., 2022). First, we transformed our effect size *r* into Fisher’s *z* and calculated its variance, because the variance of *z* depends only on sample size, unlike the sampling variance of *r*, which also depends on the effect size itself [see equations 6.2 and 6.3 in Borenstein et al., (2009)]. We then tested whether *z* depends on its associated standard error, which could indicate that small studies are more likely to be published if their effect sizes are large enough to reach statistical significance. Specifically, we ran Multilevel Linear Mixed-Effects Model with *z* as the response variable, its standard error as a fixed predictor effect, phylogenetic correction as another fixed effect (i.e., the only significant moderator; see Results section), and all random terms as described for the null model. We also tested whether publication year influenced effect sizes, which could indicate a time-lag bias (also called “bandwagon” effect), where supportive results in emerging fields are more readily published (Jennions & Moller, 2002).

## Results

**Figure 1.**
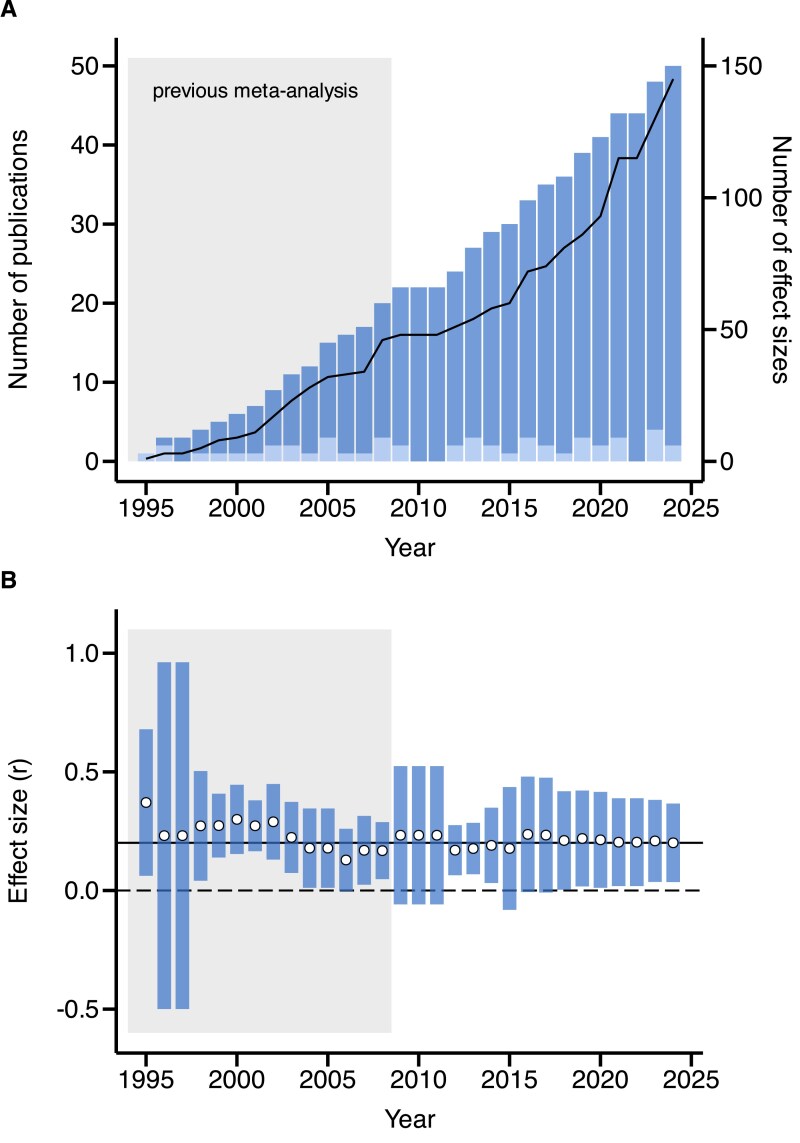
Temporal development in the field of comparative studies testing the relationship between sexual selection and speciation. Panel (A) shows the absolute (light bars) and cumulative (dark bars) number of studies published from 1995 to 2024. Solid black line denotes the cumulative number of extracted effects sizes (right *y*-axis). Panel (B) illustrates how the estimated global effect size changed during that period with the continued influx of new effect sizes. Open circles show mean global effect sizes and bars their 95% confidence intervals. Solid horizontal line indicates the final global effect size obtained after including all effect sizes. Gray areas in A and B indicate the period covered in a previous meta-analysis by Kraaijeveld et al., (2011).

We detected a largely steady publication output of comparative studies examining the relationship between sexual selection and speciation proxies over the last 30 years (Figure 1A). Primary studies encompassed a broad taxonomic range of animals, including spiders, insects, fish, amphibians, squamates, birds, and mammals, although birds were highly overrepresented and insects highly underrepresented, comprising 41% and 6% of all effect sizes, respectively (Figure S2; Table S1). This taxonomic bias was found to be persistent throughout the study period (Figure S3).

**Figure 2.**
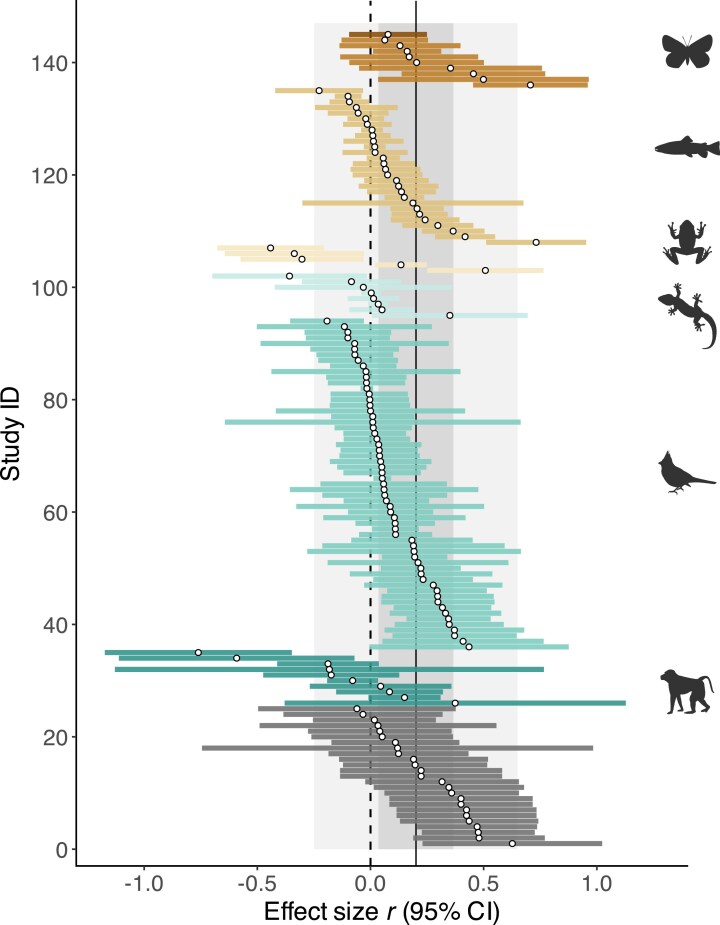
Forest plot depicting the correlation between estimates of sexual selection and speciation obtained from all primary studies. Open circles indicate Pearson correlation coefficients and bars their 95% confidence intervals. Solid vertical line indicates the estimated global effect size with the dark gray area being its 95% confidence interval and the light gray areas its 95% prediction interval. Effect sizes are ordered according to their taxonomic groups and its value. Effect sizes shown with dark gray bars originate from primary studies that encompassed multiple taxonomic classes.

Overall, we found support for a positive relationship between proxies of sexual selection and speciation, with a global effect size of *r* = 0.201 (95% confidence interval: 0.035–0.366), indicating a moderate effect (Table 1; Figure 2). Despite evidence for an overall positive effect, we detected high heterogeneity among effect sizes, primarily attributable to taxonomic affinities of effect sizes and to differences across studies (Table 1). Studies on insects, fishes, and birds seemed to find a stronger relationship between sexual selection and speciation compared to squamates and mammals, but the highly imbalanced sample size calls for great caution in interpreting this finding (Table 2; Figure S4).

**Table 1.** Results of Multilevel Linear Mixed-Effects Models testing the global effect of the relationship between sexual selection and speciation.

	Statistic	Traditional (full dataset)	Phylogenetic (full dataset)	Phylogenetic (filtered dataset)
Sample size	*N*_Studies_	50	50	49
	*k*	145	145	139
Global effect	*r*	0.126	0.201	0.183
	95% CI	(0.066, 0.186)	(0.035, 0.366)	(0.025, 0.341)
	95% PI	(−0.236, 0.488)	(−0.249, 0.650)	(−0.237, 0.603)
	*t*-value	4.159	2.400	2.297
	*P*-value	<0.001	0.018	0.023
Heterogeneity	*I* ^2^_Observation_	1.72	1.23	1.42
	*I* ^2^_Study_	67.98	35.39	32.74
	*I* ^2^_Taxon_	17.28	0	0
	*I* ^2^_Phylogeny_	–	53.54	55.03
	*I* ^2^_Total_	86.98	90.16	89.19

The traditional model ignores taxonomic affinities of effect sizes, whereas phylogenetic models account for phylogenetic non-independence. The filtered dataset excludes effect sizes based on outlier analysis and quality assessment. For all models, the total number of studies (*N*_Studies_) and effect sizes (*k*), the global effect size (*r*) with its 95% confidence intervals (CI) and 95% prediction intervals (PI), and estimates of heterogeneity (*I*²) in % are reported.

**Table 2.** Meta-regressions testing the effects of biological and methodological moderators on effect sizes (Pearson’s *r*) for the relationship between sexual selection and speciation.

Moderator	*N*	*dfs*	*Q*_M_	*P*-value
Taxonomic clade*	139	5	15.44	0.009
Speciation proxy	145	2	0.81	0.667
Sexual selection proxy	145	4	3.71	0.447
Sex-specific sexual selection	145	2	2.06	0.357
Sexual selection mechanism	145	2	1.36	0.506
Mating stage	145	2	0.57	0.752
Phylogenetic correction	145	1	21.26	< 0.001

Results of omnibus tests (*Q*_M_ statistic) from Multilevel Linear Mixed-Effects Models accounting for phylogenetic non-independence are presented.

* Phylogenetic correlation matrix was not included as a random term.

**Figure 3.**
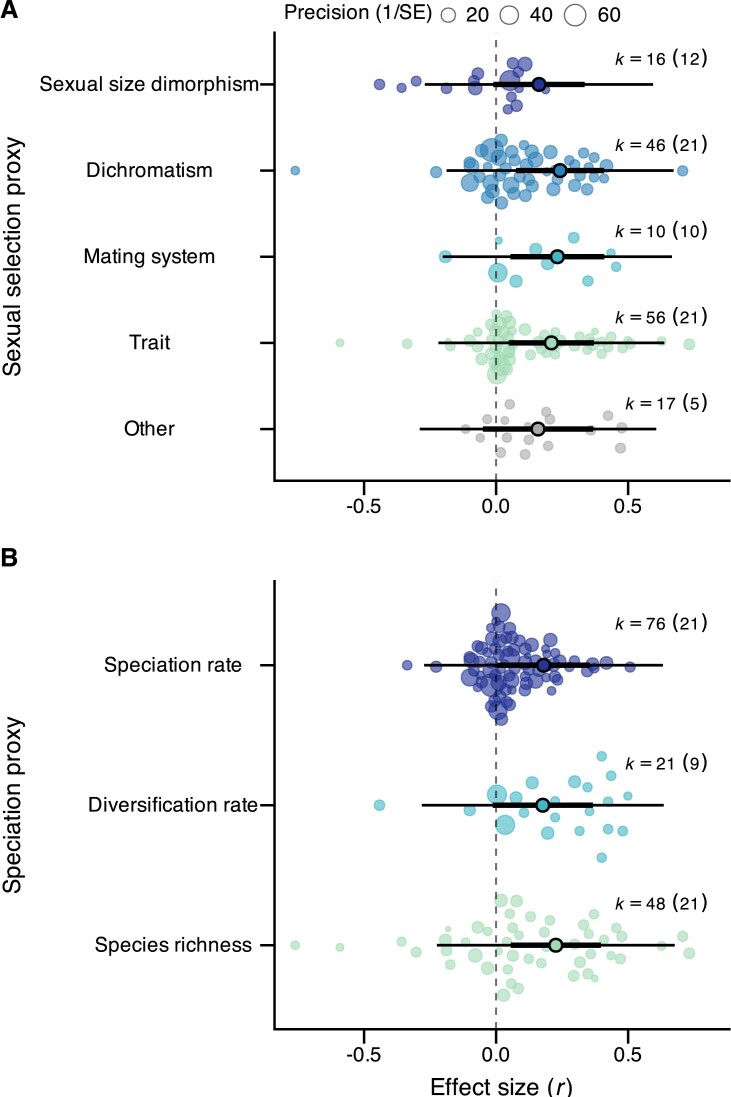
Effects of sexual selection proxy (A) and speciation proxy (B) used to estimate the correlation between sexual selection and speciation. Orchard plots show effect sizes (*k*) obtained from all primary studies (in brackets) grouped by their methodological approach. Estimated effect sizes for each group are shown with 95% confidence intervals (thick black bars) and 95% prediction intervals (whiskers).

Methodological differences between primary studies, such as the proxy used to estimate sexual selection or speciation, did not impact the observed effects (Table 2; Figure 3). Similarly, whether the sexual selection proxy focused on a particular mechanism (choice or competition), mating stage (pre-mating or post-mating), or sex (male or female) did not explain variation among the fitted effect sizes (Table 2; Figure S5A–C). In contrast, results of primary studies differed depending on whether they accounted for phylogenetic non-independence. Studies that did not statistically control for the phylogenetic relatedness of the sampled taxa reported, on average, higher effect sizes than those that did (Table 2; Figure S5D). Yet, the global effect size obtained only from studies that account for phylogenetic non-independence remained positive (*N* = 133, *r* = 0.150, *z* = 2.08, *P* = 0.037).

Sensitivity tests suggest that the aforementioned results are robust. First, our findings do not alter when using Fisher’s *z* as an alternative effect size metric instead of Pearson correlation coefficients (Tables S4 and S5). Second, a more conservative analysis, excluding six questionable effect sizes (see “Sensitivity analysis” in Methods section), supports the finding of an overall positive effect of sexual selection on speciation, though with a slightly lower global effect size (*r* = 0.183; 95% confidence interval: 0.025–0.341) (Table 1). Third, an exploration of temporal trends since the publication of the first comparative study suggests that the global effect size has remained remarkably stable following the period covered by the earlier meta-analysis by Kraaijeveld et al., (2011), despite the continued influx of new effect sizes thereafter (Figure 1B).

We did not detect signatures of publication bias in terms of a so-called small-study effect. Specifically, effect sizes of primary studies did not depend on their precision (Multilevel Linear Mixed-Effects Model, effect of standard error on *z: df* = 141, estimate ± SE = 0.346 ± 0.297, *t* = 1.168, *P* = 0.245; Figure S6A) suggesting that small studies supporting the hypothesis are not overrepresented in our dataset. Finally, we also did not observe a linear temporal change in effect sizes since the first published comparative study in 1995 (Multilevel Linear Mixed-Effects Model, effect of publication year on *z: df* = 141, estimate ± SE = 0.004 ± 0.003, *t* = 1.295, *P* = 0.198; Figure S6B).

## Discussion

Our meta-analysis provides an expanded and robust synthesis of comparative studies testing for the relationship between sexual selection and speciation across animals. By leveraging a more than two-fold larger dataset than previous efforts, we found overall evidence for a positive, albeit modest association between proxies of sexual selection and proxies of speciation. These findings offer empirical support for long-standing theoretical predictions that sexual selection drives speciation, while also uncovering persistent biological and methodological biases in research from the past three decades.

The observed global effect size (*r* = 0.201) is larger than those reported in a previous synthesis by Kraaijeveld et al., (2011), which ranged between 0.07 and 0.14, depending on the statistical approach used. However, the confidence intervals of each meta-analysis overlap with the estimated global effect size of the other, suggesting that the overall outcomes are neither qualitatively nor quantitatively different, despite the earlier studies being based on a sample size much smaller. Notably, the observed effect size corresponds to *R*² = 0.04 suggesting that on average less than 5% of the variance in speciation proxies can be explained by proxies of sexual selection. Thus, while sexual selection appears to contribute to diversification in a consistent and positive manner, our results suggest that it is not a dominant driver of speciation.

Despite an overall positive signal, we detected substantial heterogeneity in effect sizes, which could be attributed primarily to the phylogenetic signal and differences among studies, suggesting that the impact of sexual selection on speciation is context-dependent. For example, stronger relationships were typically observed in birds, fishes, and insects, whereas studies on mammals and squamates showed weaker or inconsistent patterns. These taxon-specific differences may reflect underlying biological variation in mating systems, sexual dimorphism, or ecological contexts that have been argued to modulate the impact of sexual selection on speciation (Maan & Seehausen, 2011; Mendelson & Safran, 2021; Safran et al., 2013). However, the highly unbalanced taxonomic sampling warrant caution when interpreting these patterns and highlight the need for more studies in underrepresented groups, such as insects, squamates, mammals, and others that have not yet been studied. Moreover, although sexual selection has been proposed as a potent evolutionary force in plants and fungi (Nieuwenhuis & Aanen, 2012; Tonnabel et al., 2021), our systematic literature search did not uncover a single comparative study examining its relationship with speciation in either taxon.

Interestingly, none of the methodological moderators identified in the earlier meta-analysis showed a significant signal in our study. Kraaijeveld et al., (2011) observed that studies using species numbers rather than speciation rates detected stronger signals for a positive relationship with sexual selection. Furthermore, according to Kraaijeveld et al., (2011), studies using sexual dichromatism as a proxy for the strength of sexual selection found on average stronger positive effects than those using sexual size dimorphism. We found no such effect. Neither the proxy for estimating the strength of sexual selection nor the proxy for estimating speciation explained the inter-study variation in effect sizes. This suggests that the observed positive effect is on average not dependent on the specific methodological approach used to classify or quantify sexual selection and speciation across taxonomic groups. The same holds true for biological factors such as the mechanism of sexual selection, the mating stage, or the sex in which sexual selection was tested. The only moderator explaining variation in effect sizes was whether primary studies accounted for phylogenetic non-independence, such that studies that did not control for phylogenetic effects tended to report a stronger positive effect. For instance, the early result by Barraclough et al., (1995) showed a positive correlation between sexual dichromatism and species diversity in passerine birds. However, this finding could not be replicated in later, more comprehensive studies that implemented phylogenetic correction (Cally et al., 2021; Cooney et al., 2017). Importantly, the overall positive effect obtained from our meta-analysis remained evident even when considering only studies that corrected for phylogenetic relatedness (*r* = 0.15), underscoring the robustness of the observed pattern. Accounting for statistical non-independence is likely to yield more accurate estimates of the relationship between proxies of sexual selection and speciation. However, we found that taxonomic affinities explained a significant fraction of the variation among effect sizes, suggesting that speciation events driven by sexual selection tend to be phylogenetically clustered. Consequently, applying phylogenetic correction may down-weight these conserved cases, potentially leading to a systematic underestimation of the global effect size (Uyeda et al., 2018).

The analyses of potential moderators suggests that our understanding of the biological and methodological factors driving heterogeneity among study results is limited. Even studies focusing on similar taxonomic groups or using similar methods obtained contrasting results. For instance, two studies with similar taxonomic foci used the same standardized metrics of sexual selection (i.e., Bateman gradients and opportunity for sexual selection) but different proxies for assessing speciation and different statistical approaches, did not reach the same conclusion (Janicke et al., 2018; Januario et al., 2025). Thus, the reasons for the observed between-study variation may be complex, and the moderate sample size of 50 studies, combined with the underrepresentation of certain taxonomic groups and methodological approaches, does not yet allow for testing interactions among them to explore the differing study outcomes.

We did not detect signatures of publication bias, either in terms of an overrepresentation of small studies that support the tested hypothesis or in terms of temporal linear changes in the detected effect size. Yet, beyond the above-mentioned taxonomic bias in primary studies, there is a clear imbalance among the approaches used to estimate the strength of sexual selection. The majority of primary studies used sexual dichromatism (32%) or the expression of a putative sexually selected trait (39%) as proxies for the strength of sexual selection, whereas classifications of mating systems or sexual size dimorphism have been used much less often. This imbalance is perhaps associated with a primary focus on sexual selection proxies related to mate choice rather than to competition for access to mates, reflecting a persistent bias in the field (Lackey et al., 2018; Tinghitella et al., 2018). Similarly, only 3% of the sampled comparative studies explored the effect of the strength of post-copulatory sexual selection on species diversity even though both sperm competition and cryptic female choice have been argued to facilitate reproductive isolation (Garlovsky et al., 2024; Manier et al., 2013).

An important limitation that our meta-analysis shares with all comparative studies testing for drivers of diversification lies in the difficulty of distinguishing speciation events from other processes that maintain species diversity across phylogenies. Sexual selection can not only promote speciation but may also facilitate population persistence or may contribute to population decline, thereby influencing a species’ likelihood of extinction (Rowe & Rundle, 2021; Servedio & Boughman, 2017). A large fraction of primary studies (48%) used phylogenetic data from the tested taxonomic groups to infer speciation rates. However, recent evaluations of these methods have demonstrated that phylogenies of extant species alone cannot be used to reliably disentangle speciation from extinction rates (Louca & Pennell, 2020). Thus, it remains unclear whether the positive global effect size detected by our meta-analysis reflects a true effect of sexual selection on speciation, or a more complex relationship involving a positive influence of sexual selection on species persistence.

Lastly, one might wonder whether the observed positive global effect size can be considered robust or whether it is sensitive to the moderate sample size of 50 studies, which may still leave it prone to change as the field continues to evolve. Our sensitivity analysis demonstrates that the global effect size has not changed significantly over the past 15 years, despite the continuous publication of new studies. Therefore, we do not necessarily expect a different outcome from a future meta-analysis that can capitalize on a larger sample size. Does this mean that further comparative studies testing the relationship between sexual selection and speciation are now unnecessary? In light of the persistent biases toward certain taxonomic groups and forms of sexual selection, our answer remains a clear no. Future progress in the field will critically depend on the incorporation of understudied taxa (especially invertebrates and even plants) and on exploring the role of intra-sexual competition and post-copulatory sexual selection in speciation.

In conclusion, our meta-analysis confirms a positive association between proxies of sexual selection and speciation, with notable variation across taxa and methods. However, as noted nearly two decades ago, “supporting evidence for a role of sexual selection [in speciation] is not overwhelming” (Ritchie, 2007) and limitations in current data and methodological biases underscore the need for broader taxonomic coverage and investigation of understudied sexual selection processes. Addressing these gaps in future research will be essential to fully explore the role of sexual selection in speciation.

## Supplementary Material

qraf038_Supplemental_File

## Data Availability

The data and code underlying this article are available on GitHub at https://github.com/TimJanicke/MetaAnalysis_SexSelSpec.
