# Supplementary material for: Sexual selection and speciation: a meta-analysis of comparative studies
Source: Evol Lett. 2025 Oct 22;9(6):617–27. doi: 10.1093/evlett/qraf038 (PMC12676459; doi:10.1093/evlett/qraf038)
Supplement: qraf038_Supplemental_File [file qraf038_supplemental_file.pdf]

Supplementary Material for

# Sexual selection and speciation: a meta-analysis of comparative studies

Tim Janicke[1*], Tamra C. Mendelson[2], Michael G. Ritchie[3], Lucas Marie-Orleach[4†], Jeanne Tonnabel[5†]

[*]Corresponding author

tim.janicke@cefe.cnrs.fr

[†]Equal contribution

This Supplementary Material includes

Tables S1– S5

Figures S1 – S6

Supplementary Text S1 and S2

## Supplementary Tables

**Table S1. Sample sizes for each level of the categorical moderators.** The number of studies ($N_{Studies}$), the number of effect sizes ($k$), and the proportion of effect sizes are presented for each category. Note that some primary studies reported estimates for more than one category of a given moderator.

| Moderator | Category | $N_{Studies}$ | $k$ | Proportion ($k$) |
|---|---|---|---|---|
| Taxonomic clade | Actinopterygii | 7 | 28 | 0.19 |
| | Amphibia | 3 | 5 | 0.03 |
| | Animalia | 4 | 25 | 0.17 |
| | Arachnida | 1 | 1 | 0.01 |
| | Aves | 22 | 59 | 0.41 |
| | Insecta | 6 | 9 | 0.06 |
| | Mammalia | 4 | 10 | 0.07 |
| | Squamata | 5 | 8 | 0.06 |
| Speciation proxy | Diversification rate | 9 | 21 | 0.14 |
| | Speciation rate | 21 | 76 | 0.52 |
| | Species richness | 21 | 48 | 0.33 |
| Sexual selection proxy | Dichromatism | 21 | 46 | 0.32 |
| | Mating system | 10 | 10 | 0.07 |
| | Sexual size dimorphism | 12 | 16 | 0.11 |
| | Trait | 21 | 56 | 0.39 |
| | Other | 5 | 17 | 0.12 |
| Sexual selection mechanism | Choice | 34 | 92 | 0.63 |
| | Competition | 15 | 26 | 0.18 |
| | Both | 15 | 27 | 0.19 |
| Mating stage | Post-mating | 3 | 5 | 0.03 |
| | Pre-mating | 43 | 119 | 0.82 |
| | Both | 14 | 21 | 0.14 |
| Sex | Female | 7 | 17 | 0.12 |
| | Male | 18 | 38 | 0.26 |
| | Both | 40 | 90 | 0.62 |
| Phylogenetic correction | No | 9 | 12 | 0.08 |
| | Yes | 41 | 133 | 0.92 |

**Table S2. Classification of moderators.** Table lists for each sexual selection measure used in the primary studies the number of effect sizes (*k*), along with its classification into the applied sexual selection proxy category, sexual selection mechanism, mating stage, and the sex the proxy was applied to.

| Sexual selection measure | *k* | Sexual selection proxy | Sexual selection mechanism | Mating stage | Sex |
|---|---|---|---|---|---|
| Dichromatism of breastband 1 | 1 | Dichromatism | Choice | Pre-mating | Both |
| Dichromatism of breastband 2 | 1 | Dichromatism | Choice | Pre-mating | Both |
| Dichromatism of belly | 1 | Dichromatism | Choice | Pre-mating | Both |
| Dichromatism of crown | 1 | Dichromatism | Choice | Pre-mating | Both |
| Dichromatism of mantle | 1 | Dichromatism | Choice | Pre-mating | Both |
| Dichromatism of throat | 1 | Dichromatism | Choice | Pre-mating | Both |
| Plumage dichromatism | 1 | Dichromatism | Choice | Pre-mating | Both |
| Sexual dichromatism | 36 | Dichromatism | Choice | Pre-mating | Both |
| Sexual dichromatism (colour discriminability) | 1 | Dichromatism | Choice | Pre-mating | Both |
| Sexual dichromatism (principal component) | 1 | Dichromatism | Choice | Pre-mating | Both |
| Sexual dichromatism (segment classification) | 1 | Dichromatism | Choice | Pre-mating | Both |
| Mating system | 10 | Mating system | Both | Both | Both |
| Colonial breeding | 1 | Other | Both | Both | Both |
| Female Bateman gradient (cMS[1], gMS[2]) | 1 | Other | Both | Both | Female |
| Female Bateman gradient (gMS[2]) | 1 | Other | Both | Both | Female |
| Female opportunity for sexual selection (gMS[2]) | 1 | Other | Both | Both | Female |
| Female opportunity for sexual selection (cMS1, gMS[2]) | 1 | Other | Both | Both | Female |
| Female opportunity for sexual selection (cMS[1]) | 1 | Other | Both | Pre-mating | Female |

| Sexual selection measure | *k* | Sexual selection proxy | Sexual selection mechanism | Mating stage | Sex |
|---|---|---|---|---|---|
| Male Bateman gradient (cMS[1], gMS[2]) | 1 | Other | Both | Both | Male |
| Male Bateman gradient (cMS[1]) | 1 | Other | Both | Pre-mating | Male |
| Male Bateman gradient (gMS[2]) | 1 | Other | Both | Both | Male |
| Male opportunity for sexual selection (gMS[2]) | 1 | Other | Both | Both | Male |
| Male opportunity for sexual selection (cMS[1], gMS[2]) | 1 | Other | Both | Both | Male |
| Male opportunity for sexual selection (cMS[1]) | 1 | Other | Both | Pre-mating | Male |
| Sex difference in Bateman gradient | 1 | Other | Both | Pre-mating | Both |
| Sex difference in opportunity for selection | 1 | Other | Both | Pre-mating | Both |
| Sex difference in opportunity for sexual selection | 1 | Other | Both | Both | Both |
| Sexual conflict trait | 1 | Other | Both | Both | Both |
| Spermathecal width | 1 | Other | Competition | Post-mating | Female |
| Dimorphism of size and fin | 1 | Sexual size dimorphism | Competition | Pre-mating | Both |
| Male-biased sexual selection | 1 | Sexual size dimorphism | Both | Pre-mating | Both |
| Sexual length dimorphism | 1 | Sexual size dimorphism | Competition | Pre-mating | Both |
| Sexual mass dimorphism | 1 | Sexual size dimorphism | Competition | Pre-mating | Both |
| Sexual size dimorphism | 12 | Sexual size dimorphism | Competition | Pre-mating | Both |
| Bioluminescent courtship | 1 | Trait | Choice | Pre-mating | Both |
| Dewlap size | 1 | Trait | Choice | Pre-mating | Male |
| Dimorphism of canine | 1 | Trait | Competition | Pre-mating | Both |
| Female bill colour | 1 | Trait | Choice | Pre-mating | Female |
| Female choice traits | 1 | Trait | Choice | Pre-mating | Female |
| Female colour complexity | 1 | Trait | Choice | Pre-mating | Female |
| Female extent of ornamental colours | 1 | Trait | Choice | Pre-mating | Female |

| Sexual selection measure | *k* | Sexual selection proxy | Sexual selection mechanism | Mating stage | Sex |
|---|---|---|---|---|---|
| Female maximum plumage saturation | 1 | Trait | Choice | Pre-mating | Female |
| Female mean plumage saturation | 1 | Trait | Choice | Pre-mating | Female |
| Female plumage achromatic difference | 1 | Trait | Choice | Pre-mating | Female |
| Female plumage carotenoids | 1 | Trait | Choice | Pre-mating | Female |
| Female plumage colouration | 1 | Trait | Choice | Pre-mating | Female |
| Female plumage melanin | 1 | Trait | Choice | Pre-mating | Female |
| Female-female competition traits | 1 | Trait | Competition | Pre-mating | Female |
| Male bill colour | 1 | Trait | Choice | Pre-mating | Male |
| Male choice traits | 1 | Trait | Choice | Pre-mating | Male |
| Male colour complexity | 1 | Trait | Choice | Pre-mating | Male |
| Male colouration | 1 | Trait | Choice | Pre-mating | Male |
| Male courtship | 1 | Trait | Choice | Pre-mating | Male |
| Male extent of ornamental colours | 1 | Trait | Choice | Pre-mating | Male |
| Male maximum plumage saturation | 1 | Trait | Choice | Pre-mating | Male |
| Male mean plumage saturation | 1 | Trait | Choice | Pre-mating | Male |
| Male ornamentation | 2 | Trait | Choice | Pre-mating | Male |
| Male plumage achromatic difference | 1 | Trait | Choice | Pre-mating | Male |
| Male plumage carotenoids | 1 | Trait | Choice | Pre-mating | Male |
| Male plumage colouration | 1 | Trait | Choice | Pre-mating | Male |
| Male plumage melanin | 1 | Trait | Choice | Pre-mating | Male |
| Male-male competition traits | 1 | Trait | Competition | Pre-mating | Male |
| Physical combat | 1 | Trait | Choice | Pre-mating | Male |
| Plumage brilliance | 1 | Trait | Choice | Pre-mating | Male |

| Sexual selection measure | *k* | Sexual selection proxy | Sexual selection mechanism | Mating stage | Sex |
|---|---|---|---|---|---|
| Plumage chroma | 1 | Trait | Choice | Pre-mating | Male |
| Plumage colour span | 1 | Trait | Choice | Pre-mating | Male |
| Plumage colour volume | 1 | Trait | Choice | Pre-mating | Male |
| Plumage hue disparity | 1 | Trait | Choice | Pre-mating | Male |
| Presence of auditory traits | 1 | Trait | Choice | Pre-mating | Both |
| Presence of bright genitalia | 1 | Trait | Choice | Pre-mating | Both |
| Presence of derived melanosomes | 1 | Trait | Choice | Pre-mating | Both |
| Presence of follicular glands | 2 | Trait | Choice | Pre-mating | Male |
| Presence of gustatory traits | 1 | Trait | Choice | Pre-mating | Both |
| Presence of olfactory traits | 1 | Trait | Choice | Pre-mating | Both |
| Presence of sexual swellings | 1 | Trait | Choice | Pre-mating | Both |
| Presence of sexually selected traits | 1 | Trait | Choice | Pre-mating | Both |
| Presence of tactile traits | 1 | Trait | Choice | Pre-mating | Both |
| Presence of visual traits | 1 | Trait | Choice | Pre-mating | Both |
| Presence of weaponry | 2 | Trait | Competition | Pre-mating | Both |
| Residual testes weight | 1 | Trait | Competition | Post-mating | Male |
| Song note type, pace and number | 1 | Trait | Choice | Pre-mating | Male |
| Song pitch and bandwidth | 1 | Trait | Choice | Pre-mating | Male |
| Spermatophore count | 1 | Trait | Competition | Post-mating | Male |
| Territorial call | 1 | Trait | Competition | Pre-mating | Male |
| Testis length | 1 | Trait | Competition | Post-mating | Male |
| Testis size | 1 | Trait | Competition | Post-mating | Male |
| Wing pigmentation | 1 | Trait | Choice | Pre-mating | Male |

[1]estimated based on copulatory mating success; [2]estimated based on genetic mating success

**Table S3. Effect sizes excluded in conservative analysis testing for global effect of sexual selection on speciation.** Table lists for each study the number of excluded effect sizes and the applied exclusion criterion.

| Study | Number of effect sizes excluded | Reason for exclusion |
|---|---|---|
| Gage MJG, Parker GA, Nylin S, Wiklund C, 2002. Sexual selection and speciation in mammals, butterflies and spiders. Proc R Soc B-Biol Sci 269:2309-2316. | 1 | Low sample size ($N < 10$) |
| Januario M, Macedo-Rego RC, Rabosky DL, 2024. Evolutionary lability of sexual selection and its implications for speciation and macroevolution. Am Nat. doi: 10.1086/734457. | 1 | Low sample size ($N < 10$) |
| Misof B, 2002. Diversity of Anisoptera (*Odonata*): Infering speciation processes from patterns of morphological diversity. Zoology 105:355-365. | 1 | High Cook's distance ($D_i = 0.057$) |
| Nunn CL, Altizer S, Sechrest W, Jones KE, Barton RA, Gittleman JL, 2004. Parasites and the evolutionary diversification of primate clades. Am Nat 164: S90-S103. | 1 | Failed to pass outlier test (Grubbs test: $G = 3.91$, $P = 0.004$) |
| Nunn CL, Altizer S, Sechrest W, Jones KE, Barton RA, Gittleman JL, 2004. Parasites and the evolutionary diversification of primate clades. Am Nat 164: S90-S103. | 2 | Low sample size ($N < 10$) |
| Total | 6 | |

**Table S4. Results of Multilevel Linear Mixed-Effects Models testing for a global effect of sexual selection on speciation using Fisher's *z* as effect size.** The traditional model ignores phylogenetic affinities of effect sizes, whereas phylogenetic models account for phylogenetic non-independence. The filtered dataset excludes effect sizes based on outlier analysis and quality assessment. For all models, the number of studies ($N_{Studies}$) and effect sizes ($k$), the global effect size (Fisher's $z$) with its 95% confidence intervals (CI) and 95% prediction interval (PI), and estimates of heterogeneity ($I^2$) in % are reported.

| | Statistic | Traditional (full dataset) | Phylogenetic (full dataset) | Phylogenetic (filtered dataset) |
|---|---|---|---|---|
| Sample size | $N_{Studies}$ | 50 | 50 | 49 |
| | $k$ | 145 | 145 | 139 |
| Global effect | $z$ | 0.105 | 0.171 | 0.159 |
| | 95% CI | (0.053, 0.156) | (0.027, 0.314) | (0.019, 0.298) |
| | 95% PI | (-0.183, 0.392) | (-0.209, 0.550) | (-0.209, 0.526) |
| | $t$-value | 4.035 | 2.354 | 2.255 |
| | $P$-value | < 0.001 | 0.020 | 0.026 |
| Heterogeneity | $I^2_{Observation}$ | 2.02 | 1.43 | 1.57 |
| | $I^2_{Study}$ | 53.63 | 33.81 | 33.74 |
| | $I^2_{Taxon}$ | 24.38 | 0 | 0 |
| | $I^2_{Phylogeny}$ | – | 50.86 | 50.46 |
| | $I^2_{Total}$ | 80.04 | 86.11 | 85.77 |

**Table S5. Meta-regressions testing the effects of biological and methodological moderators on effect sizes (Fisher's *z*) for the relationship between sexual selection and speciation.** Results of omnibus tests ($Q_M$ statistic) from Multilevel Linear Mixed-Effects Models accounting for phylogenetic non-independence are presented.

| Moderator | *N* | *dfs* | $Q_M$ | *P*-value |
|---|---|---|---|---|
| Taxonomic clade* | 139 | 5 | 13.38 | 0.020 |
| Speciation proxy | 145 | 2 | 0.14 | 0.932 |
| Sexual selection proxy | 145 | 4 | 4.06 | 0.398 |
| Sex-specific sexual selection | 145 | 2 | 1.48 | 0.477 |
| Sexual selection mechanism | 145 | 2 | 2.84 | 0.242 |
| Mating stage | 145 | 2 | 0.42 | 0.809 |
| Phylogenetic correction | 145 | 1 | 14.93 | < 0.001 |

*Phylogenetic correlation matrix was not included as a random term.

## Supplementary Figures

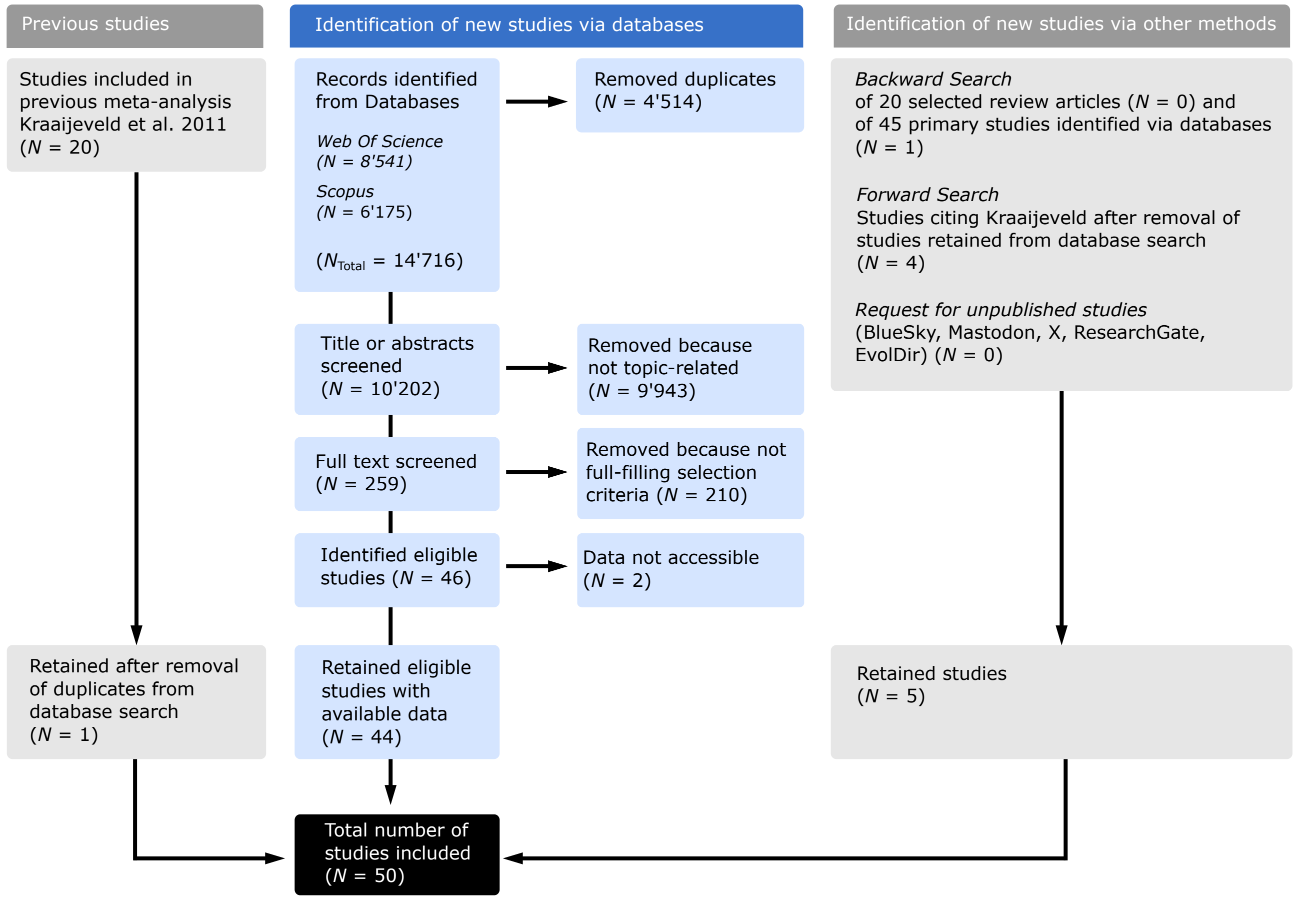


**Figure S1. PRISMA diagram.** Flow chart indicating how primary studies of this meta-analysis have been identified and selected.

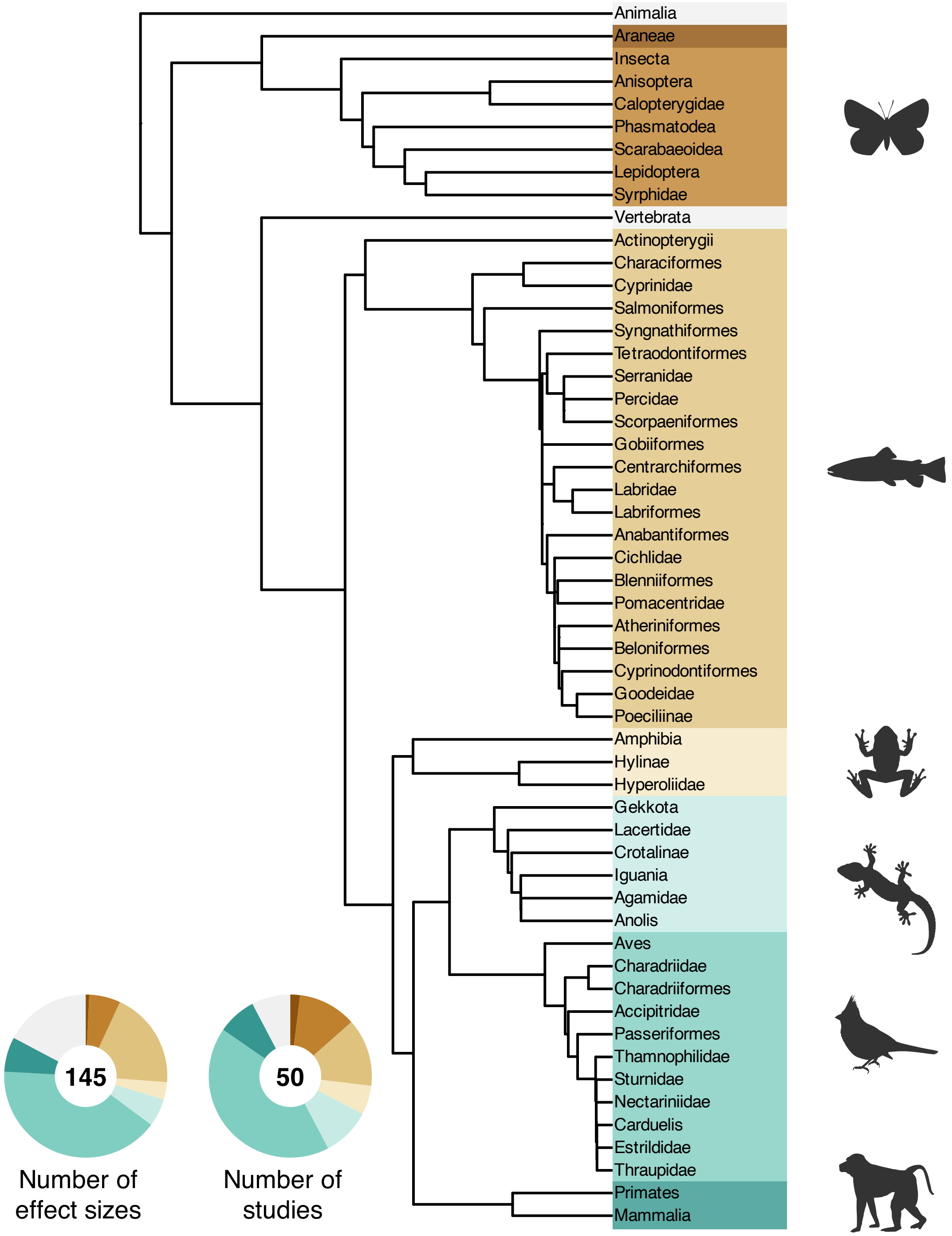


**Figure S2. Phylogeny of taxonomic groups studied by primary studies.** Doughnut charts indicate the proportion of effect sizes and studies sampled for each taxonomic class. This time-calibrated tree was used to account for phylogenetic non-independence.

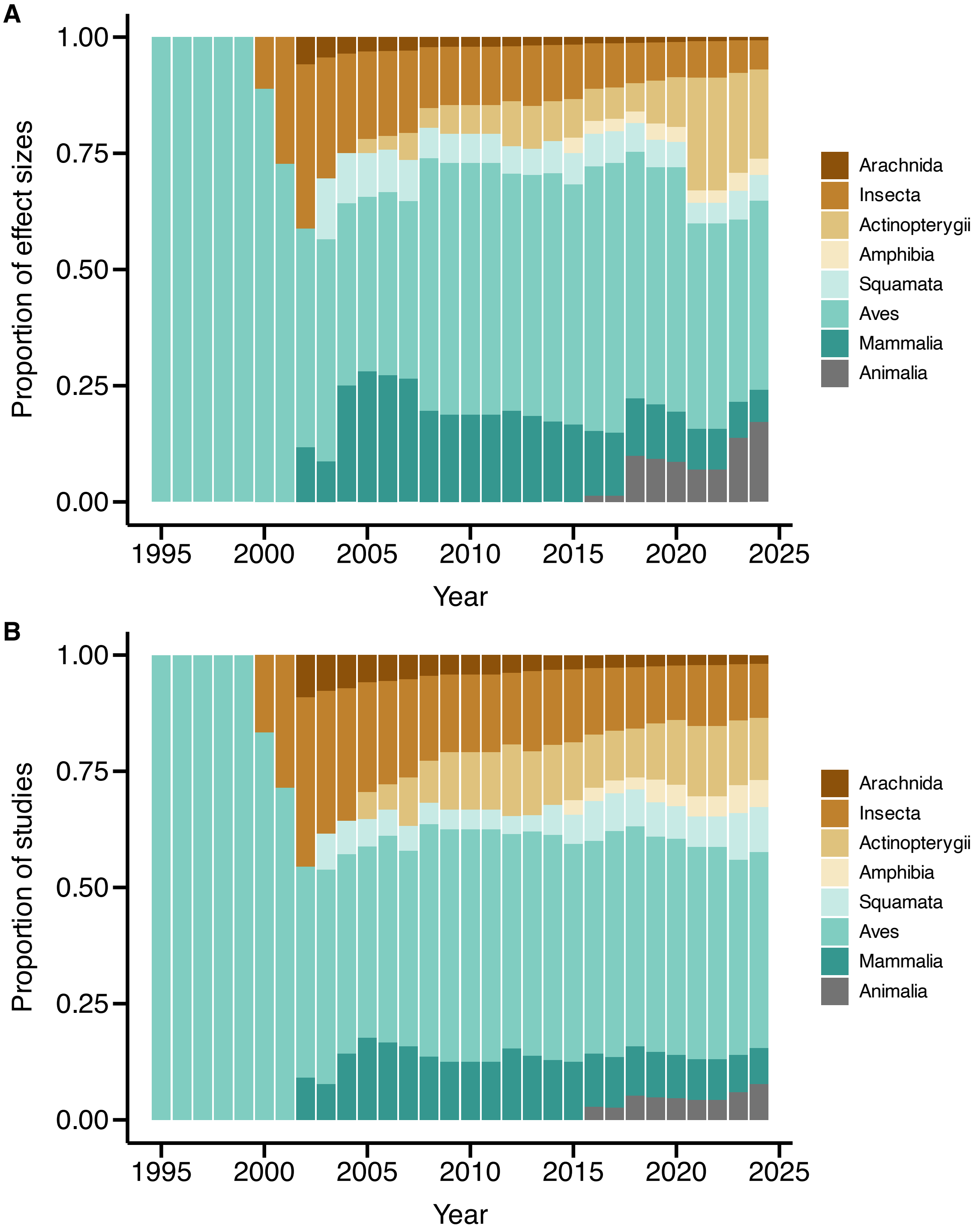


**Figure S3. Temporal changes in taxonomic bias of primary studies included in the meta-analysis.** Stacked bars show the proportion of each sampled major taxonomic clade based on cumulative data on the number of (A) effect sizes and (B) studies.

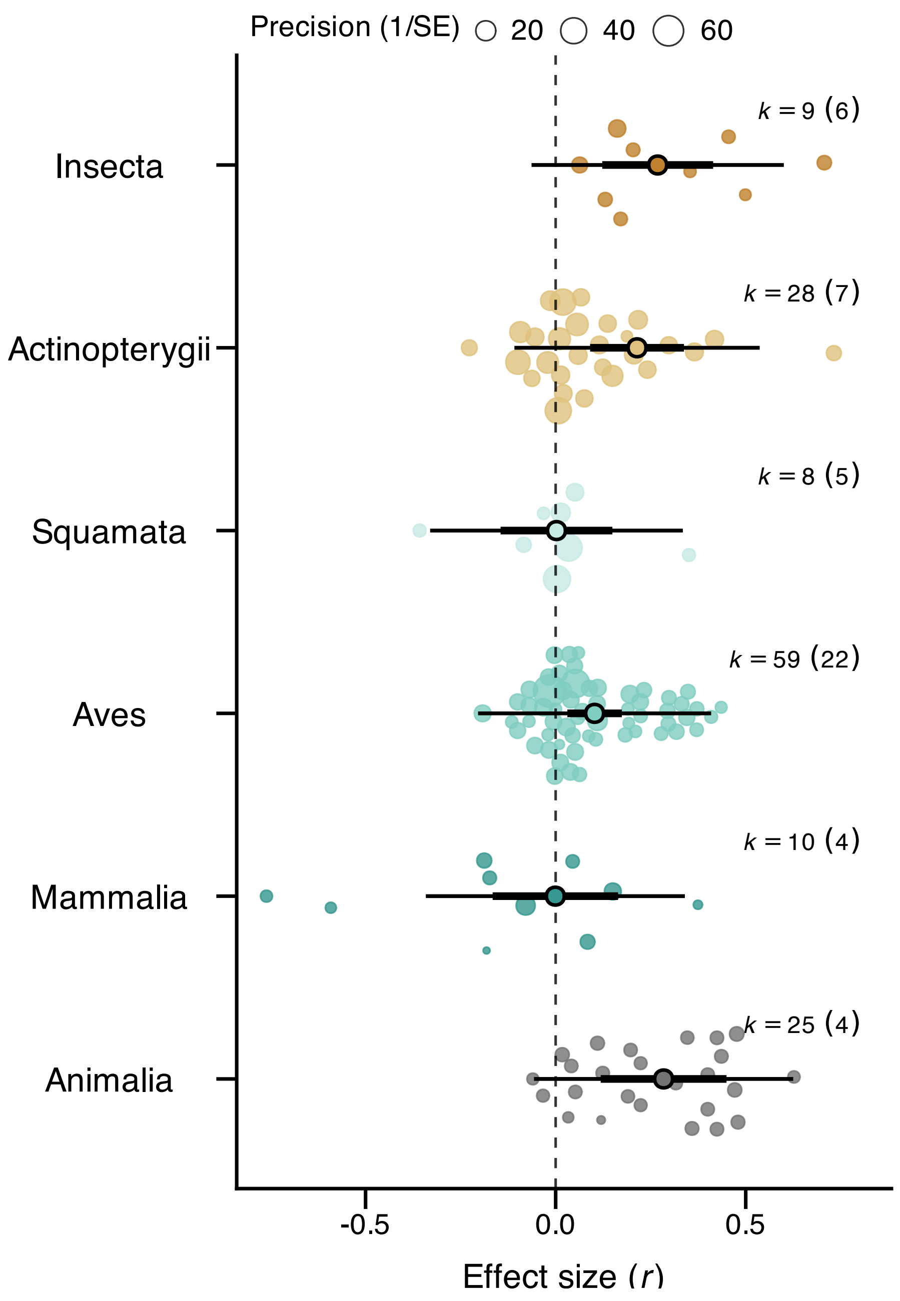


**Figure S4. Comparison of effect sizes measuring the correlation between sexual selection and speciation across major taxonomic clades.** Orchard plots show effect sizes ($k$) obtained from all primary studies (in brackets) grouped by their taxonomic clade. Estimated effect sizes for each group are shown with 95% confidence intervals (thick black bars) and 95% prediction intervals (whiskers). Only clades with more than five effect sizes are plotted.

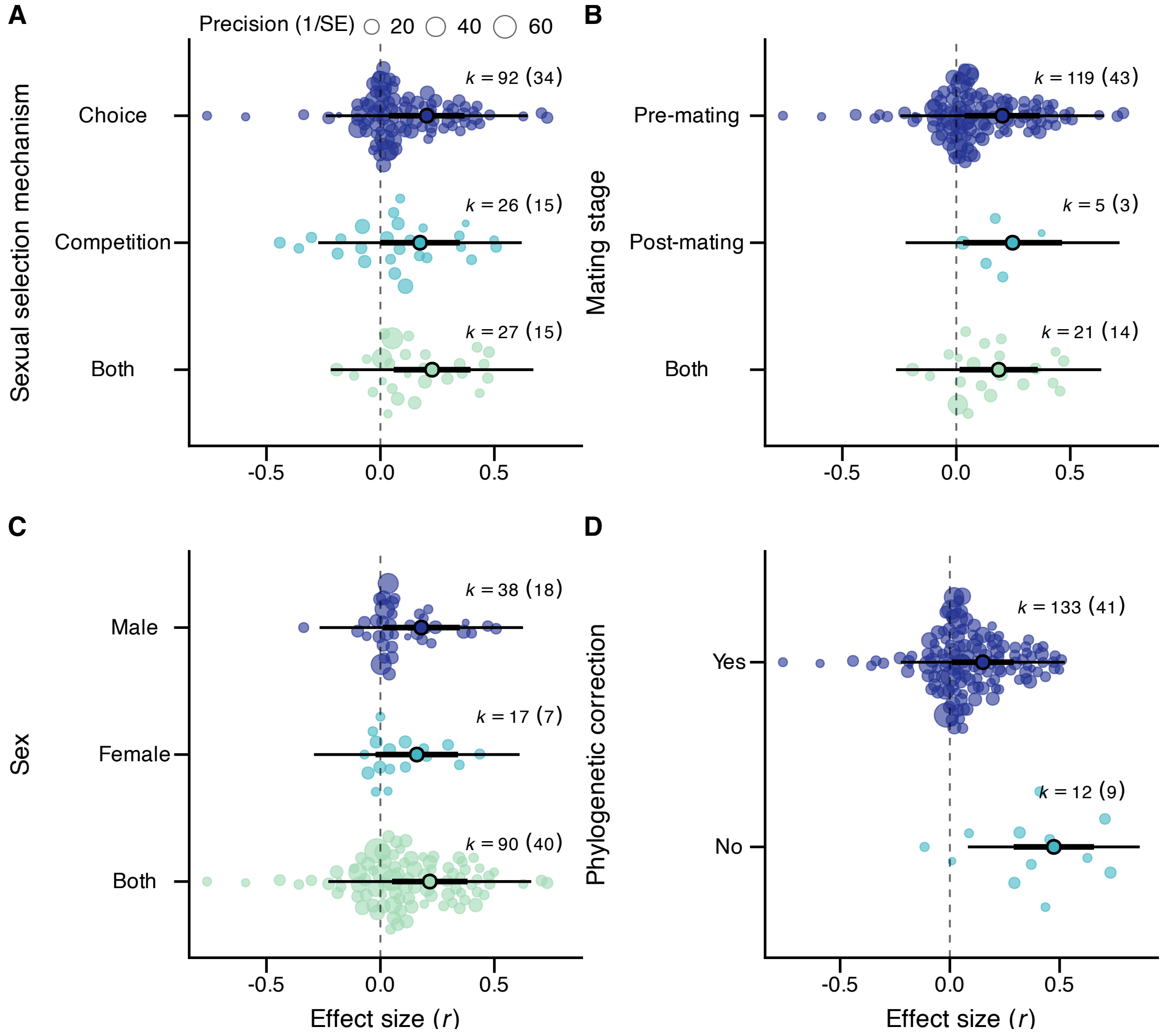


**Figure S5. Effect of moderators on effect sizes measuring the correlation between sexual selection and speciation across major taxonomic clades.** Orchard plots show the effect of the tested (A) sexual selection mechanism, (B) the mating stage, (C) the tested sex, and (D) whether the primary study applied phylogenetic correction on all sampled effect sizes (*k*) obtained from all primary studies (in brackets). Estimated effect sizes for each group are shown with 95% confidence intervals (thick black bars) and 95% prediction intervals (whiskers).

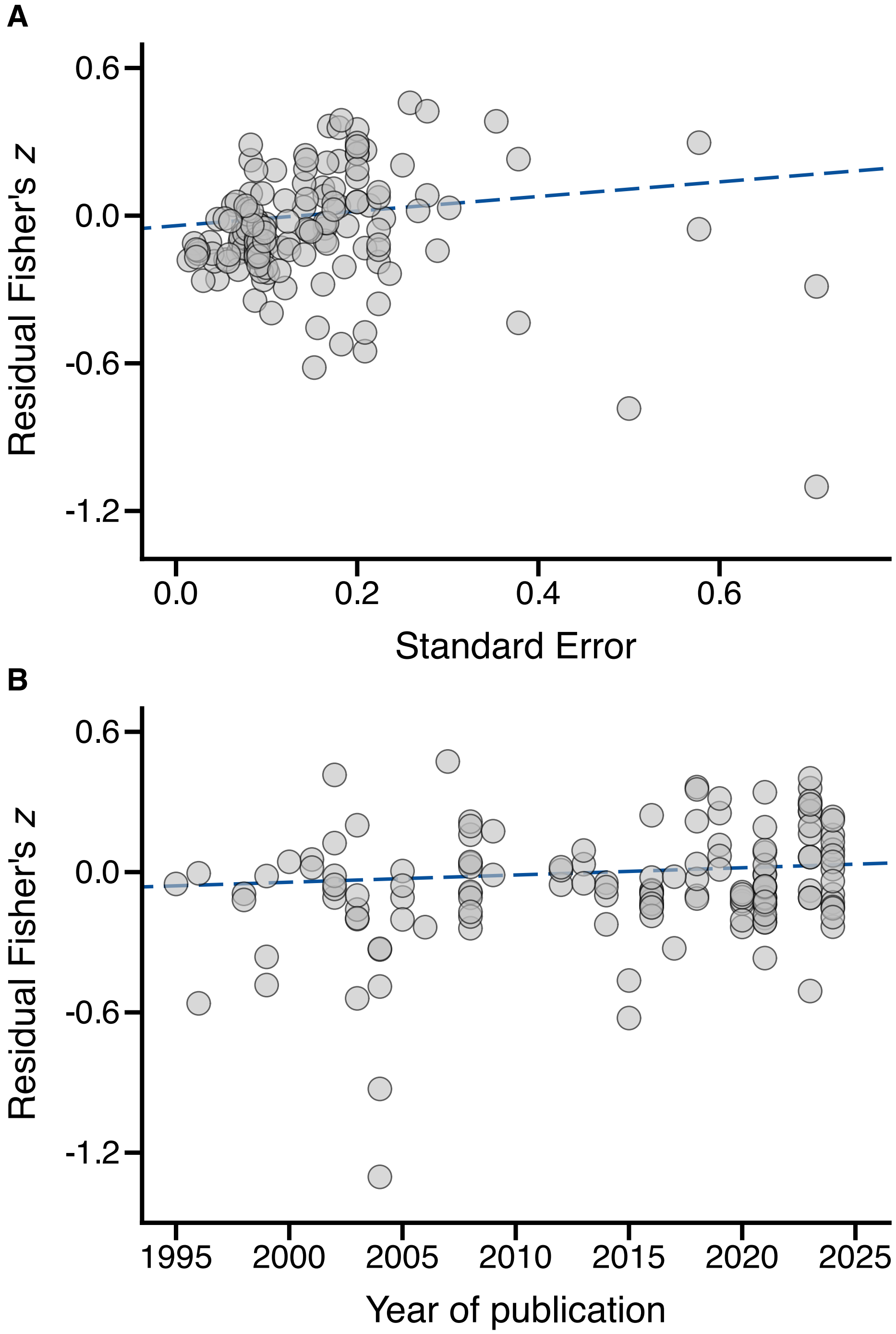


**Figure S6. Exploration of publication bias in terms of (A) small study effect and (B) time-lag bias.** The residuals of Fisher's *z*, derived from the full Multilevel Linear Mixed-Effects Models, are plotted against (A) the standard error of Fisher's *z* and (B) the year of publication. The dashed blue line represents the estimated slope.

## Supplementary Texts

**Supplementary Text S1.** Full references of the 20 influential reviews used in the backward search for primary studies.


Bonduriansky R, 2011. Sexual selection and conflict as engines of ecological diversification. Am Nat 178:729-745. doi: 10.1086/662665.

Boughman JW, Brand JA, Brooks RC, Bonduriansky R, Wong BBM, 2024. Sexual selection and speciation in the Anthropocene. Trends Ecol Evol 39:654-665. doi: 10.1016/j.tree.2024.02.005.

Dijkstra PD, Border SE, 2018. How does male-male competition generate negative frequency-dependent selection and disruptive selection during speciation? Current Zoology 64:89-99. doi: 10.1093/cz/zox079.

Grether GF, Breed M, Moore J, 2010. Sexual selection and speciation. Encyclopedia of animal behavior Academic, Oxford:177-183.

Haghighatnia M, Machac A, Schmickl R, Placette CL, 2023. Darwin's 'mystery of mysteries': the role of sexual selection in plant speciation. Biol Rev 98:1928-1944. doi: 10.1111/brv.12991.

Lackey ACR, Martin MD, Tinghitella RM, 2018. Male competition and speciation: expanding our framework for speciation by sexual selection. Current Zoology 64:69-73. doi: 10.1093/cz/zoy009.

Lackey ACR, Scordato ESC, Keagy J, Tinghitella RM, Heathcote RJP, 2024. The role of mate competition in speciation and divergence: a systematic review. J Evol Biol 37:1225-1243. doi: 10.1093/jeb/voae111.

Lipshutz SE, 2018. Interspecific competition, hybridization, and reproductive isolation in secondary contact: missing perspectives on males and females. Current Zoology 64:75-88. doi: 10.1093/cz/zox060.

Maan ME, Seehausen O, 2011. Ecology, sexual selection and speciation. Ecol Lett 14:591-602. doi: 10.1111/j.1461-0248.2011.01606.x.

Mendelson TC, Safran RJ, 2021. Speciation by sexual selection: 20 years of progress. Trends Ecol Evol 36:1153-1163. doi: 10.1016/j.tree.2021.09.004.

Panhuis TM, Butlin R, Zuk M, Tregenza T, 2001. Sexual selection and speciation. Trends Ecol Evol 16:364-371. doi: 10.1016/s0169-5347(01)02160-7.

Price T, 1998. Sexual selection and natural selection in bird speciation. Philos Trans R Soc B-Biol Sci 353:251-260. doi: 10.1098/rstb.1998.0207.

Questiau S, 1999. How can sexual selection promote population divergence? Ethol Ecol Evol 11:313-324. doi: 10.1080/08927014.1999.9522816.

Ritchie MG, 2007. Sexual selection and speciation. Annu Rev Ecol Evol Syst 38:79-102. doi: 10.1146/annurev.ecolsys.38.091206.095733.

Safran RJ, Scordato ESC, Symes LB, Rodríguez RL, Mendelson TC, 2013. Contributions of natural and sexual selection to the evolution of premating reproductive isolation: a research agenda. Trends Ecol Evol 28:643-650. doi: 10.1016/j.tree.2013.08.004.

Scordato ESC, Symes LB, Mendelson TC, Safran RJ, 2014. The role of ecology in speciation by sexual Selection: A systematic empirical review. J Hered 105:782-794. doi: 10.1093/jhered/esu037.

Servedio MR, Boughman JW, 2017. The role of sexual selection in local adaptation and speciation. Annual Review of Ecology, Evolution, and Systematics, Vol 48 48:85-109. doi: 10.1146/annurev-ecolsys-110316-022905.

Tinghitella RM, Lackey ACR, Martin M, Dijkstra PD, Drury JP, Heathcote R, Keagy J, Scordato ESC, Tyersi AM, 2018. On the role of male competition in speciation: a review and research agenda. Behav Ecol 29:783-797. doi: 10.1093/beheco/arx151.

Tsuji K, Fukami T, 2020. Sexual dimorphism and species diversity: from clades to sites. Trends Ecol Evol 35:105-114. doi: 10.1016/j.tree.2019.09.001.

Uy JAC, Irwin DE, Webster MS, 2018. Behavioral isolation and incipient speciation in birds. Annual Review of Ecology, Evolution, and Systematics, Vol 49 49:1-24. doi: 10.1146/annurev-ecolsys-110617-062646.

**Supplementary Text S2.** Full references of all 50 primary studies in alphabetical order.


Alfaro ME, Brock CD, Banbury BL, Wainwright PC, 2009. Does evolutionary innovation in pharyngeal jaws lead to rapid lineage diversification in labrid fishes? BMC Evol Biol 9:14. doi: 10.1186/1471-2148-9-255.

Arnqvist G, Edvardsson M, Friberg U, Nilsson T, 2000. Sexual conflict promotes speciation in insects. Proc Natl Acad Sci U S A 97:10460-10464. doi: 10.1073/pnas.97.19.10460.

Barraclough TG, Harvey PH, Nee S, 1995. Sexual selection and taxonomic diversity in passerine birds. Proc R Soc B-Biol Sci 259:211-215. doi: 10.1098/rspb.1995.0031.

Cally JG, Stuart-Fox D, Holman L, Dale J, Medina I, 2021. Male-biased sexual selection, but not sexual dichromatism, predicts speciation in birds. Evolution 75:931-944. doi: 10.1111/evo.14183.

Cardoso GC, Mota PG, 2008. Speciational evolution of coloration in the genus *Carduelis*. Evolution 62:753-762. doi: 10.1111/j.1558-5646.2008.00337.x.

Cooney CR, Tobias JA, Weir JT, Botero CA, Seddon N, 2017. Sexual selection, speciation and constraints on geographical range overlap in birds. Ecol Lett 20:863-871. doi: 10.1111/ele.12780.

D'Urban Jackson J, dos Remedios N, Maher KH, Zefania S, Haig S, Oyler-McCance S, Blomqvist D, Burke T, Bruford MW, Székely T, Küpper C, 2017. Polygamy slows down population divergence in shorebirds. Evolution 71:1313-1326. doi: 10.1111/evo.13212.

De Lisle SP, Rowe L, 2015. Independent evolution of the sexes promotes amphibian diversification. Proc R Soc B Biol Sci 282. doi: 10.1098/rspb.2014.2213.

de Solan T, Sinervo B, Geniez P, David P, Crochet PA, 2023. Colour polymorphism and conspicuousness do not increase speciation rates in Lacertids. Peer Community J 3:18. doi: 10.24072/pcjournal.345.

Ellis EA, Oakley TH, 2016. High rates of species accumulation in animals with bioluminescent courtship displays. Curr Biol 26:1916-1921. doi: 10.1016/j.cub.2016.05.043.

Emberts Z, Wiens JJ, 2021. Do sexually selected weapons drive diversification? Evolution 75:2411-2424. doi: 10.1111/evo.14212.

FitzJohn RG, Maddison WP, Otto SP, 2009. Estimating trait-dependent speciation and extinction rates from incompletely resolved phylogenies. Syst Biol 58:595-611. doi: 10.1093/sysbio/syp067.

Furness AI, Pollux BJA, Meredith RW, Springer MS, Reznick DN, 2019. How conflict shapes evolution in poeciliid fishes. Nat Commun 10:12. doi: 10.1038/s41467-019-11307-5.

Gage MJG, Parker GA, Nylin S, Wiklund C, 2002. Sexual selection and speciation in mammals, butterflies and spiders. Proc R Soc B-Biol Sci 269:2309-2316. doi: 10.1098/rspb.2002.2154.

Gomes ACR, Sorenson MD, Cardoso GC, 2016. Speciation is associated with changing ornamentation rather than stronger sexual selection. Evolution 70:2823-2838. doi: 10.1111/evo.13088.

Hendry CR, Guiher TJ, Pyron RA, 2014. Ecological divergence and sexual selection drive sexual size dimorphism in new world pitvipers (Serpentes: *Viperidae*). J Evol Biol 27:760-771. doi: 10.1111/jeb.12349.

Huang H, Rabosky DL, 2014. Sexual selection and diversification: Reexamining the correlation between dichromatism and speciation rate in birds. Am Nat 184:E101-E114. doi: 10.1086/678054.

Iglesias-Carrasco M, Jennions MD, Ho SYW, Duchêne DA, 2019. Sexual selection, body mass and molecular evolution interact to predict diversification in birds. Proc R Soc B-Biol Sci 286:7. doi: 10.1098/rspb.2019.0172.

Ingram T, Harrison A, Mahler DL, Castañeda MDR, Glor RE, Herrel A, Stuart YE, Losos JB, 2016. Comparative tests of the role of dewlap size in Anolis lizard speciation. Proc R Soc B Biol Sci 283. doi: 10.1098/rspb.2016.2199.

Isaac NJB, Jones KE, Gittleman JL, Purvis A, 2005. Correlates of species richness in mammals: Body size, life history, and ecology. Am Nat 165:600-607. doi: 10.1086/429148.

Janicke T, Ritchie MG, Morrow EH, Marie-Orleach L, 2018. Sexual selection predicts species richness across the animal kingdom. Proc R Soc B-Biol Sci 285:8. doi: 10.1098/rspb.2018.0173.

Januario M, Macedo-Rego RC, Rabosky DL, 2024. Evolutionary lability of sexual selection and its implications for speciation and macroevolution. Am Nat. doi: 10.1086/734457.

Katzourakis A, Purvis A, Azmeh S, Rotheray G, Gilbert F, 2001. Macroevolution of hoverflies (Diptera: *Syrphidae*): the effect of using higher-level taxa in studies of biodiversity, and correlates of species richness. J Evol Biol 14:219-227. doi: 10.1046/j.1420-9101.2001.00278.x.

Krüger O, 2008. Engines of speciation: A comparative study in birds of prey. J Evol Biol 21:861-872. doi: 10.1111/j.1420-9101.2008.01502.x.

Luría-Manzano R, Pinheiro PDP, Kohlsdorf T, Haddad CFB, Martins M, 2023. Evolution of territoriality in Hylinae treefrogs: Ecological and morphological correlates and lineage diversification. J Evol Biol 36:1090-1101. doi: 10.1111/jeb.14189.

Magnuson-Ford K, Otto SP, 2012. Linking the investigations of character evolution and species diversification. Am Nat 180:225-245. doi: 10.1086/666649.

Maia R, Rubenstein DR, Shawkey MD, 2013. Key ornamental innovations facilitate diversification in an avian radiation. Proc Natl Acad Sci U S A 110:10687-10692. doi: 10.1073/pnas.1220784110.

Mank JE, 2007. Mating preferences, sexual selection and patterns of cladogenesis in ray-finned fishes. J Evol Biol 20:597-602. doi: 10.1111/j.1420-9101.2006.01251.x.

McGee MD, Borstein SR, Meier JI, Marques DA, Mwaiko S, Taabu A, Kishe MA, O'Meara B, Bruggmann R, Excoffier L, Seehausen O, 2020. The ecological and genomic basis of explosive adaptive radiation. Nature 586:75-+. doi: 10.1038/s41586-020-2652-7.

Miller EC, Mesnick SL, Wiens JJ, 2021. Sexual dichromatism Is decoupled from diversification over deep time in fishes. Am Nat 198:232-252. doi: 10.1086/715114.

Misof B, 2002. Diversity of Anisoptera (*Odonata*): Infering speciation processes from patterns of morphological diversity. Zoology 105:355-365. doi: 10.1078/0944-2006-00076.

Mitra S, Landel H, PruettJones S, 1996. Species richness covaries with mating system in birds. Auk 113:544-551.

Moller AP, Cuervo JJ, 1998. Speciation and feather ornamentation in birds. Evolution 52:859-869. doi: 10.1111/j.1558-5646.1998.tb03710.x.

Mooers AO, Moller AP, 1996. Colonial breeding and speciation in birds. Evol Ecol 10:375-385. doi: 10.1007/bf01237724.

Morrow EH, Pitcher TE, Arnqvist G, 2003. No evidence that sexual selection is an 'engine of speciation' in birds. Ecol Lett 6:228-234. doi: 10.1046/j.1461-0248.2003.00418.x.

Murali G, Meiri S, Roll U, 2023. Chemical signaling glands are unlinked to species diversification in lizards. Evolution 77:1829-1841. doi: 10.1093/evolut/qpad101.

Nicolaï MPJ, Van Hecke B, Rogalla S, Debruyn G, Bowie RCK, Matzke NJ, Hackett SJ, D'Alba L, Shawkey MD, 2024. The evolution of multiple color mechanisms is correlated with diversification in sunbirds (*Nectariniidae*). Syst Biol 73:343–354. doi: 10.1093/sysbio/syae006.

Nunn CL, Altizer S, Sechrest W, Jones KE, Barton RA, Gittleman JL, 2004. Parasites and the evolutionary diversification of primate clades. Am Nat 164:S90-S103. doi: 10.1086/424608.

Owens IPF, Bennett PM, Harvey PH, 1999. Species richness among birds: Body size, life history, sexual selection or ecology? Proc R Soc B Biol Sci 266:933-939. doi: 10.1098/rspb.1999.0726.

Phillimore AB, Freckleton RP, Orme CDL, Owens IPF, 2006. Ecology predicts large-scale patterns of phylogenetic diversification in birds. Am Nat 168:220-229. doi: 10.1086/505763.

Portik DM, Bell RC, Blackburn DC, Bauer AM, Barratt CD, Branch WR, Burger M, Channing A, Colston TJ, Conradie W, Dehling JM, Drewes RC, Ernst R, Greenbaum E, Gvozdik V, Harvey J, Hillers A, Hirschfeld M, Jongsma GFM,

Kielgast J, Kouete MT, Lawson LP, Leache AD, Loader SP, Lotters S, Van Der Meijden A, Menegon M, Muller S, Nagy ZT, Ofori-Boateng C, Ohler A, Papenfuss TJ, Roessler D, Sinsch U, Roedel MO, Veith M, Vindum J, Zassi-Boulou AG, McGuire JA, 2019. Sexual dichromatism drives diversification within a major radiation of african amphibians. Syst Biol 68:859-875. doi: 10.1093/sysbio/syz023.

Price-Waldman RM, Shultz AJ, Burns KJ, 2020. Speciation rates are correlated with changes in plumage color complexity in the largest family of songbirds. Evolution 74:1155-1169. doi: 10.1111/evo.13982.

Ritchie MG, Webb SA, Graves JA, Magurran AE, Garcia CM, 2005. Patterns of speciation in endemic Mexican Goodeid fish: sexual conflict or early radiation? J Evol Biol 18:922-929. doi: 10.1111/j.1420-9101.2005.00919.x.

Seddon N, Botero CA, Tobias JA, Dunn PO, MacGregor HEA, Rubenstein DR, Uy JAC, Weir JT, Whittingham LA, Safran RJ, 2013. Sexual selection accelerates signal evolution during speciation in birds. Proc R Soc B-Biol Sci 280:9. doi: 10.1098/rspb.2013.1065.

Seddon N, Merrill RM, Tobias JA, 2008. Sexually selected traits predict patterns of species richness in a diverse clade of suboscine birds. Am Nat 171:620-631. doi: 10.1086/587071.

Sol D, Stirling DG, Lefebvre L, 2005. Behavioral drive or behavioral inhibition in evolution: Subspecific diversification in holarctic passerines. Evolution 59:2669-2677. doi: 10.1111/j.0014-3820.2005.tb00978.x.

Stuart-Fox D, Owens IPF, 2003. Species richness in agamid lizards: chance, body size, sexual selection or ecology? J Evol Biol 16:659-669. doi: 10.1046/j.1420-9101.2003.00573.x.

Svensson EI, Waller JT, 2013. Ecology and sexual selection: Evolution of wing pigmentation in calopterygid damselflies in relation to latitude, sexual dimorphism, and speciation. Am Nat 182:E174-E195. doi: 10.1086/673206.

Tuschhoff E, Wiens JJ, 2023. Evolution of sexually selected traits across animals. Front Ecol Evol 11:11. doi: 10.3389/fevo.2023.1042747.

Wagner CE, Harmon LJ, Seehausen O, 2012. Ecological opportunity and sexual selection together predict adaptive radiation. Nature 487:366-369. doi: 10.1038/nature11144.
